# Natural Antimicrobial Nano Composite Fibres Manufactured from a Combination of Alginate and Oregano Essential Oil

**DOI:** 10.3390/nano11082062

**Published:** 2021-08-13

**Authors:** Hao Lu, Jonathan A. Butler, Nicole S. Britten, Prabhuraj D. Venkatraman, Sameer S. Rahatekar

**Affiliations:** 1 Enhanced Composites and Structures Centre, School of Aerospace, Transport and Manufacturing, Cranfield University, Bedfordshire MK43 0AL, UK; hao.lu@cranfield.ac.uk; 2 Department of Life Sciences, Faculty of Science and Engineering, Manchester Metropolitan University, Chester Street, Manchester M1 5GD, UK; jonathan.butler@mmu.ac.uk (J.A.B.); nicole.s.britten@stu.mmu.ac.uk (N.S.B.); 3 Manchester Fashion Institute, Faculty of Arts and Humanities, Manchester Metropolitan University, Cavendish Street, Manchester M15 6BH, UK

**Keywords:** electrospinning, nanofibres, sodium alginate, oregano oil, antimicrobial resistance

## Abstract

Alginate is a linear biodegradable polysaccharide polymer, which is bio-renewable and widely used for various biomedical applications. For the next generation of medical textiles, alginate nanofibres are desirable for their use in wound dressings that are biocompatible, sustainable, and abundantly available. This study has developed a unique manufacturing process for producing alginate nanofibres with exceptional antimicrobial properties of oregano essential oil (OEO) as a natural antimicrobial agent. OEO with varying degrees of concentration was incorporated in an aqueous alginate solution. Appropriate materials and electrospinning process parameter selection allowed us to manufacture alginate fibres with a range of diameters between 38 and 105 nm. A unique crosslinking process for alginate nanofibres using extended water soaking was developed. Mechanical characterisation using micro-mechanical testing of nonwoven electrospun alginate/oregano composite nanofibres revealed that it was durable. An extensive antimicrobial study was carried out on alginate/oregano composite nanofibres using a range of Gram-positive (methicillin-resistant *Staphylococcus aureus* (MRSA) and *Listeria monocytogenes*) and Gram-negative bacteria (*Klebsiella pneumoniae* and *Salmonella enterica*), which are common wound and food pathogens. The results indicated that increasing the concentration of OEO from 2 to 3 wt % showed improved antimicrobial activity against all pathogens, and activity was significantly improved against MRSA compared to a non-alginate-based control disk containing OEO. Therefore, our research suggests that all-natural alginate/oregano nanofibre composite textiles offer a new generation of medical textiles for advanced wound dressing technology as well as for food packaging applications.

## 1. Introduction

With the advancement of polymer science and chemistry, there has been a tremendous demand for ultra-fine fibres (nanofibres) for varied applications, particularly for biomedical applications, drug delivery systems, bone tissue engineering [1,2,3], tissue regeneration, and wound dressing technology [4]. Electrospinning enables the production of fibres with diameters of 100 nm or less [5] that have a larger surface area to volume ratio and are highly porous [6,7]. A typical electrospinning apparatus consists of a high voltage supply, flow control pump, a spinneret, and a collector [7]. During the electrospinning process, high voltage is applied to produce an electrically charged jet of polymer solution directed by electrostatic forces resulting in an interconnected fibrous membrane [8]. The polymer solution that is extruded from the spinneret is electrified to generate a jet, followed by stretching and elongation to generate fibres [6]. Initially, the jet from the spinneret extends in a straight line and then undergoes instability where it extends to form a conical shaped jet; finally, the jet stretches and solidifies and deposits onto the grounded collector. Various process parameters affect the properties of the electrospun fibrous membrane, including precursor solution (conductivity, surface tension, viscosity), processing parameters (voltage applied, flow rate, and distance between nozzle and collector), and ambient conditions (temperature and humidity) [8,9]. When gathered on a stationary collector, electrospun fibres form a porous and random structure that resembles the 3D architecture of collagen fibres found in the skin extracellular matrix [10,11]. Recently, there has been a surge toward the need for biodegradable polymers for medical applications.

**Figure 1:**
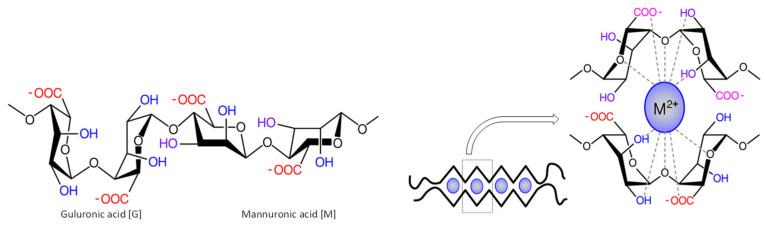
Structure of alginate and egg-box model with guluronic and mannuronic acid.

Alginate is a biodegradable polymer derived from brown seaweed and is readily soluble in water [12]. Alginate, also called algin or alginic acid, is composed of 1–4-linked *β*–D-mannuronic and *α*–L–guluronic acid residues [13] obtained from cell walls of brown algae [13]. Alginate acid residues are arranged in egg-box-like structures (Figure 1) and are composed of linear polysaccharides with monomeric acid residues linked together in different compositions and have a polyelectrolytic tendency inadequate for electrospinning [6,14]. Hence, alginate has been blended with hydrosoluble polymers (polyvinyl alcohol (PVA) and polyethylene oxide (PEO)) to become electro-spinnable and to produce bead-free fibres [13]. Alginate has unique properties, including being easily mouldable, biodegradable, biocompatible, renewable, and abundantly available, making it an ideal biomaterial for biomedical applications [13]. Recent advances have resulted in an increase in the consumption of alginate for a range of applications. The global alginate consumption for wound care applications is predicted to grow by 4.2% CAGR (compounded annual growth rate) from 2016 to 2025 and is expected to reach $923.8 million by 2025 [15].

Antimicrobial resistance is a major global issue [16], and mortality rates associated with antimicrobial-resistant infections are predicted to exceed 10 million people per annum by 2050 [17]. The development of novel systems for delivering antimicrobial agents to sites of infection/contamination is one strategy for combatting antimicrobial resistance. Although alginate has excellent functional characteristics, it does not possess natural antimicrobial properties against microorganisms. Hence, it becomes essential to blend or combine it with suitable constituents that inhibit microbial growth. Oregano essential oil (OEO) is an effective natural and sustainable antimicrobial agent obtained from plant species derived from *Origanum* (family: Lamiaceae) and *Lippia* (family: *Verbenaceae*) [18,19].

Plant-based components such as clove, coriander, cinnamon, thyme, mint, rosemary, mustard, and cilantro, in addition to OEO, possess natural antimicrobial properties and are being further investigated for biomedical applications [20]. Carvacrol and thyme are the main compounds identified within OEO, which provide a distinct odour and antimicrobial and antioxidant activity [20]. Preus et al. (2005) studied the antimicrobial efficacy of several aromatic oils and reported that OEO was potent against two bacterial strains of *Staphylococcus aureus* termed ATCC 14154 and ATCC 14775 [21]. The fungicidal properties of OEO have also been reported [22]. Plant-based antimicrobial compounds possess several advantages over synthetic chemicals as they are often non-toxic, have limited side effects, and are environmentally friendly [23]. Alginate films with varying concentrations of OEO (0.5–1.5 wt %) were characterised for their mechanical and antimicrobial properties. Crosslinking of alginate films with calcium carbonate increased the tensile strength and had low elongation. Researchers reported that a minimum concentration of 1% was required to show antimicrobial efficacy. Alginate films with OEO were more effective against Gram-positive bacteria (*S. aureus and L. monocytogenes*) than Gram-negative bacteria (*E. coli and S. enteritidis*) [24]. Sodium alginate, PEO, and curcumin-based composite nanofibres were also crosslinked with biocompatible trifluoroacetic acid with improved mechanical properties [25]. Therefore, it could be noted that crosslinking improves the mechanical properties of alginate.

As alginate is challenging to extrude via electrospinning, in this study, sodium alginate was blended with PEO solution, and OEO was uniformly added to produce an electrospun solution resulting in an ultra-fine nanofibre composite. Physical properties of the resulting nanofibres were examined, and the antimicrobial efficacy against the major wound and foodborne infection-causing bacterial pathogens was characterised, and suitable biomedical and consumer applications were identified. This research contributes to the literature, particularly in the electrospinning and manufacturing of all-natural antimicrobial alginate nanofibres composites, which has advantages over synthetic antimicrobial compounds that are harmful to the environment. Various process parameters have been modified, and the best possible method has been described to produce nanofibre morphology incorporated with OEO.

## 2. Materials and Methods

### 2.1. Reagents

Alginate acid sodium salt from brown algae (SA, medium viscosity, SIGMA A2033, Sigma-Aldrich, MO, USA), polyethylene oxide (PEO, MW 1,000,000, Alfa Aesar, Haverhill, MA, USA) powder (Pluronic^®^ F-127, PL, SIGMA P2443, Sigma-Aldrich, MO, USA), calcium chloride powder (Fisher Scientific, Loughborough, UK) and oregano essential oil (OEO) were used to produce electrospun nanofibres. Oregano essential vulgare oil (100%) was obtained from Athina Greece, with its main constituents comprised of carvacrol, 80%; thymol, 4.2%; p-cymol, 4.2%; gamma-terpinen, 1.6%; limonen, 0.22%; sabinen, 0.25%; and linalool, 0.12%; the rest of the components were less than 0.1%.

### 2.2. Solution Preparation

Alginate electrospun fibres were prepared using the following methods. First, 3.75 wt % of sodium alginate and 4.0 wt % PEO solutions were prepared individually in deionised water with continuous stirring at 20 °C temperature for 24 to 36 h until complete dissolution. Sodium alginate and PEO solution were then mixed in a ratio of 80:20. Pluronic^®^ F-127 at 1 wt % and OEO at 2 and 3 wt % of the whole solution were further added and incubated at approximately 50 °C with stirring for 24 h to reach complete and uniform dissolution. The solution was placed in a vacuum oven and removed once no bubbles were seen in the solution.

#### Viscosity Measurements

The viscosity measurements were carried out using a Rheometer AR2000ex (TA Instruments, Crawley, UK). The viscosity measurements were carried out at room temperature (25 °C) using a parallel plate arrangement (plate size 40 mm) with shear rate ranging from 1 s^−1^ to 200 s^−1^.

### 2.3. Electrospinning

Prior to the electrospinning process, the syringe with target solution, catheter, and needle were connected, and tin foil was placed on the collector for sample collection. An LE-50 FLUIDNATEK™ Electro Spinning tool (Bioinicia, Valencia, Spain) was used for electrospinning experiments for a duration of approximately 8 h, with the main parameters set as follows: 1) the diameter of the needle was 0.4 mm; 2) the distance between needle and collector was kept at 15 cm; 3) both the board collector and rotator were used to collect the sample with the rotator speed set to 600–900 rpm; 4) the voltage was applied as 24–30 kV while the feed rate of the syringe stayed at 0.8–1.2 mL/h; 5) all spinning processes were performed at room temperature (18–25 °C) and humidity of 40–60%.

**Figure 2:**
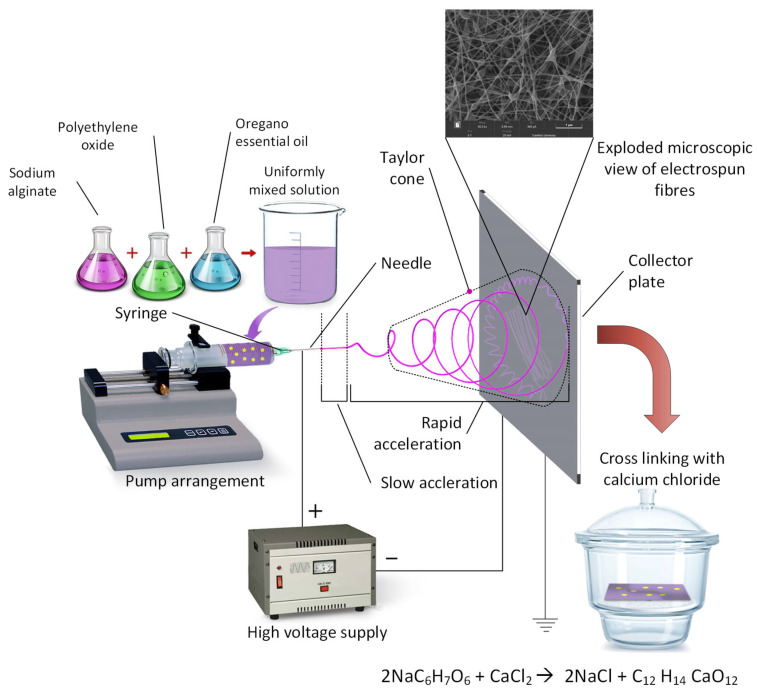
Simple schematic diagram of electrospinning process. The formation of Taylor cones and transition zone can be clearly seen at the tip of the needle (rapid acceleration) and crosslinking process.

The conditions of the electrospinning and the solution viscosity differed; hence, the setting parameters for electrospinning were always presented as a range. The Taylor cone formed in the spinning tip of the needle and the transition zone (the zone with rapid acceleration, which is between the liquid discharged from the needle and the fibre formation) were examined periodically, which was also to determine if the electrospinning process was carried out correctly. The voltage and feed rate were adjusted until a stable Taylor cone and conversion area were formed (Figure 2).

### 2.4. Crosslinking Process

**Figure 3:**
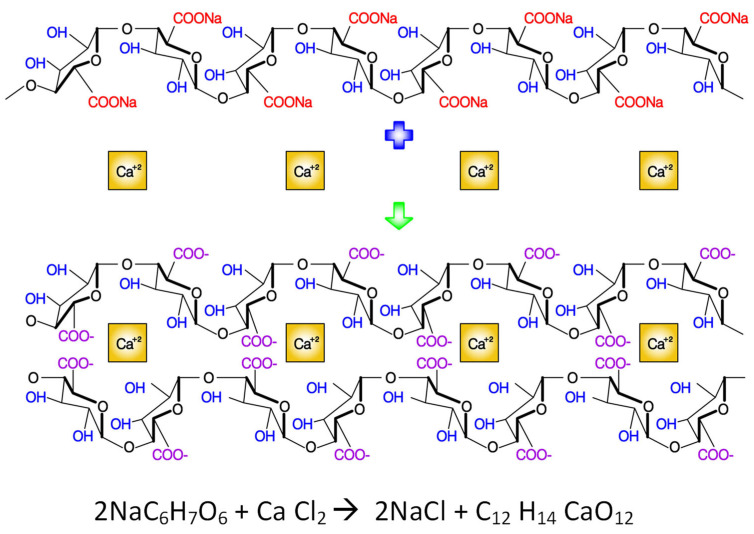
Crosslinking of sodium alginate with calcium chloride.

Crosslinking of alginate with calcium chloride improves the mechanical properties, particularly tensile strength, due to a crosslinking reaction between Ca^2+^ ions and carboxyl groups of sodium alginate [26]. Alginate polymer consists of blocks containing two uronic acids and two chain-forming heteropolysaccharides made up of blocks *β–*(1–4) linked D mannuronic (M) and *α*–(1–4) linked L–guluronic acids. Their structure varies depending on the monomer position forming homopolymeric or heteropolymeric blocks. The physical and mechanical properties of alginate gels depend on the relative proportion of blocks [26]. In this study, oregano oil was blended within the fibre. There were some changes in the crosslinking process and had two main steps. First, the electrospun fibre samples were removed from the tin foil collector, and 4 wt % calcium chloride solution with deionised water was prepared. The samples were immersed in calcium chloride for 10 min, soaked in deionised water for 24 h, and dried in the open air. Figure 3 illustrates the typical crosslinking process of sodium alginate with calcium chloride.

The ionic crosslinking of sodium alginate is a well-established process as reported in the literature [27,28]. Further to the above-mentioned studies, Liling et al. [29] showed that sodium alginate films achieved complete ionic crosslinking by immersing the films in 2 wt % calcium chloride for 2 min. The completion of the crosslinking process was confirmed by the distinct increase in the mechanical properties of the ionically crosslinked films compared to non-crosslinked sodium alginate films. Similarly, Ibrahim et al. [26] showed the ionic crosslinking process of sodium alginate films by immersing it in calcium chloride for 2–8 min; their ionic crosslinking process also confirmed superior mechanical properties (tensile strength and tensile modulus).

### 2.5. Analysis of Physical Properties

#### 2.5.1. Scanning Electron Microscopy (SEM)

SEM was used to measure the diameter of the nanofibres. Samples were analysed using an S8000 Environmental Scanning Electron Microscope (ESEM) (TESCAN, Kohoutovice, Czech Republic). Samples were coated with AU10 (gold) prior to analysis using an electron beam of 15–20 keV. Micrographs were analysed using Photoshop with samples evenly divided into 25 grids to measure the diameter of the fibres. The diameter of nanofibres were measured by counting the fibres between the grids using ‘Image J’ software (Java based image processing program, National Institutes of Health, University of Wisconsin, USA) and the scale bar from the micrographs.

#### 2.5.2. Mechanical Test

**Figure 4:**
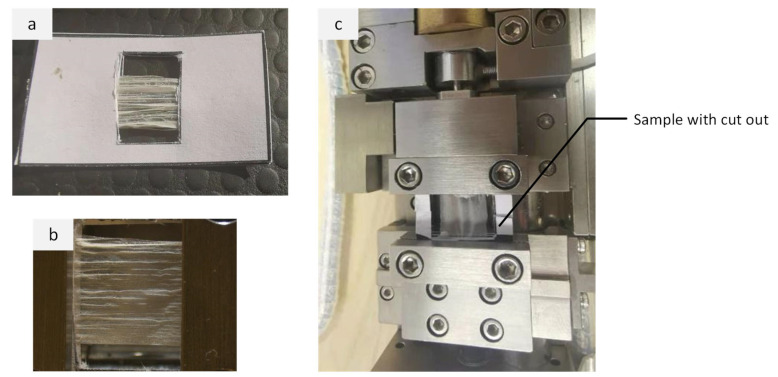
(**a**) Electrospun sample sandwiched between the rectangular plates 10mm wide, (**b**) close-up view of the sample, and (**c**) sample placed between the upper and lower jaws of the tensile equipment.

A DEBEN 200N Microtest Tensile Stage Controller (Suffolk, UK) was used to measure the tensile strength of fibres. Samples were slowly stretched with a minor force, slowly rising until breakage to determine the tensile strength and deformation. For the tensile test, the sample was sandwiched between two square plates with 10 mm wide rectangular cut-outs to ensure the sample was aligned such that stretch was carried out in the lengthwise direction of the fibres and to maintain stability (Figure 4).

### 2.6. Antimicrobial Susceptibility

#### 2.6.1. Bacterial Strains

Methicillin-resistant *Staphylococcus aureus* strain USA300 (ST8) JE2, *Pseudomonas aeruginosa* strain PAO1, *Salmonella enterica* serotype Typhimurium strain 14028, and *Listeria monocytogenes* strain Scott A were cultured using Mueller Hinton (MH) agar (Oxoid, Basingstoke, UK) and incubated under aerobic conditions at 37 °C for 24 h.

#### 2.6.2. Contact Assay

Contact assays were performed using a modified Deutsches Institut für Normung (DIN) 58940-3 agar diffusion standard method [30,31]. Briefly, 6 mm diameter circular discs of each respective sample was added to MH agar plates pre-inoculated with bacterial cultures, which were adjusted to a McFarland standard of 0.5. Inoculated agar plates were incubated for 24 h at 37 °C under aerobic conditions, and bacterial growth inhibitory zones were determined using a digital calliper gauge. Filter paper discs (6 mm diameter; Whatman, Cytiva, Buckinghamshire, UK) with 3% final concentration Oregano oil (EssentialOils Online, Norfolk, UK) were included as a control. All assays were performed in triplicate with biological replicates (n = 3).

#### 2.6.3. Statistical Analysis

One-way ANOVA with Tukey’s statistical tests for post hoc analysis were performed using GraphPad Prism version 8.4.2 (GraphPad Software, San Diego, CA, USA).

## 3. Results and Discussion

It is worth noting that during the electrospinning process many changes have taken place to ensure optimal conditions were generated to produce alginate nanofibres. Sample S1 was produced initially using the above process and is discussed later in the section. Table 1 shows the various samples produced in this study using various manufacturing process parameters.

### 3.1. The Influence of Adding OEO

**Figure 5:**
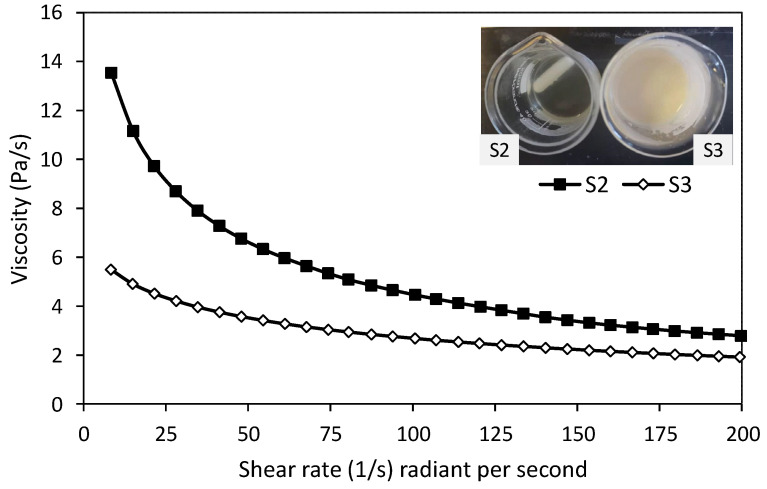
Solution without OEO (inset S2) and solution with OEO (inset S3). Viscosity of solution without OEO (line with dark squares, S2) and solution with Oregano (line with diamond shape, S3).

To determine the effect of adding OEO to the target solution (alginate/PEO solution) on the spinning process, solution S2 (without OEO) and solution S3 (with 2% OEO) were prepared. It was noted that the solution without OEO was a transparent yellow colour, while the solution with OEO was more turbid than the solution without OEO (Figure 5, inset S2 and S3). The viscosity of solutions S2 and S3 was examined, as shown in Figure 5, where OEO reduced the viscosity of the solution (Figure 5, line with diamond). In this study, electrospinning was carried out using a voltage of 28 kV; the distance between the needle and plate was 15 cm, at room temperature.

**Figure 6:**
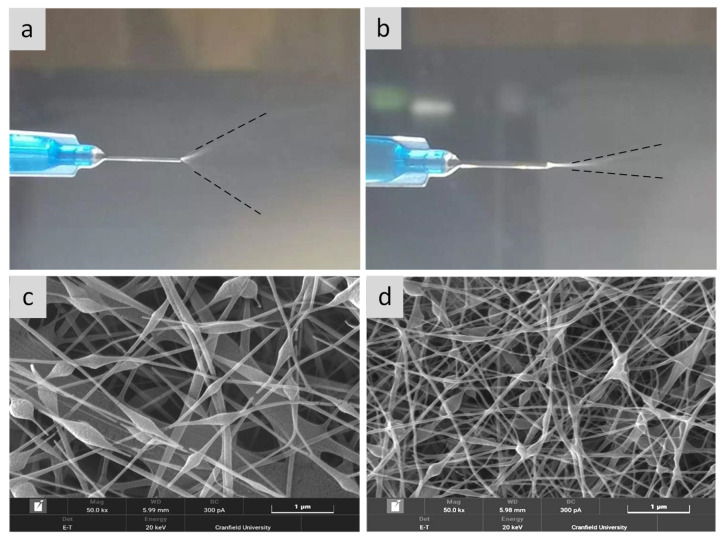
Influence of OEO towards density of electrospun fibres: (**a**) spinneret showing fibre jet with the spinning solution without OEO and (**b**) with OEO; (**c**) the microscopic image of fibre mat without OEO (S2); (**d**) the microscopic image of fibre mat with OEO (S3).

According to Figure 6a,b, when a stable Taylor cone was formed, it can be clearly seen that the transition zone and spread angle of S2 were significantly larger than those of S3. SEM images revealed formation of nanofibres with larger beads in S2 (Figure 6c) compared to sample S3 (Figure 6d). Adding OEO into alginate and PEO solution reduced the viscosity of the solution, resulting in nanofibres with smaller bead formation.

### 3.2. The Influence of Voltage on Fibre Morphology

In the electrospinning process, the influence of voltage affects the structural morphology of nanofibres [5]. An increase in voltage from 16 kV to 25 kV produced nanofibres [poly (d, L–Lactic acid)] with varying fibre morphology. The diameter of the beads was smaller when compared to low voltage, and the average distance between the beads was shorter at 25 kV. Any increase in the voltage produced nanofibres with a large diameter and changed the shape of the beads (spindle to spherical shaped) [32]. Previous research on sodium alginate produced cylindrical nanofibres (90 ± 20 nm) when maintaining a voltage of 17 kV, flow rate of 0.8 mL/h, and tip to collector distance of 17 cm [33].

**Figure 7:**
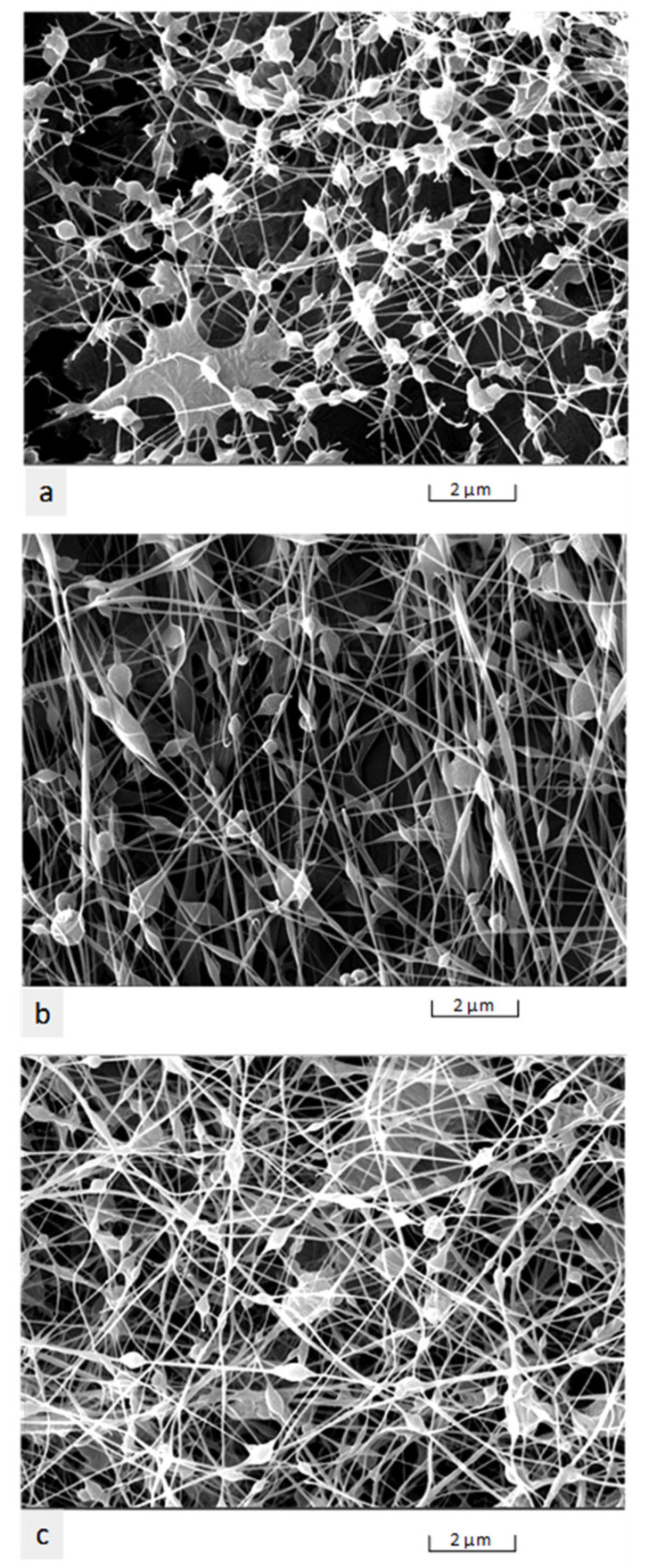
SEM micrographs—the influence of voltage on fibre morphology: (**a**) 22 kV; (**b**) 26 kV; and (**c**) 30 kV.

Interestingly, in this study at 22 kV, we observed that the nanofibres were spaced out with smaller beads (Figure 7a). At 26 kV, micrographs (Figure 7b) revealed that the nanofibres were denser, with distinct spindle-shaped beads; however, at 30 kV, the nanofibres were more prominent in diameter with a smaller size of beads, and the average distance between the beads was smaller (Figure 7c). These findings were in agreement with a previous study [32].

### 3.3. The Influence of Different Crosslinking Processes

**Figure 8:**
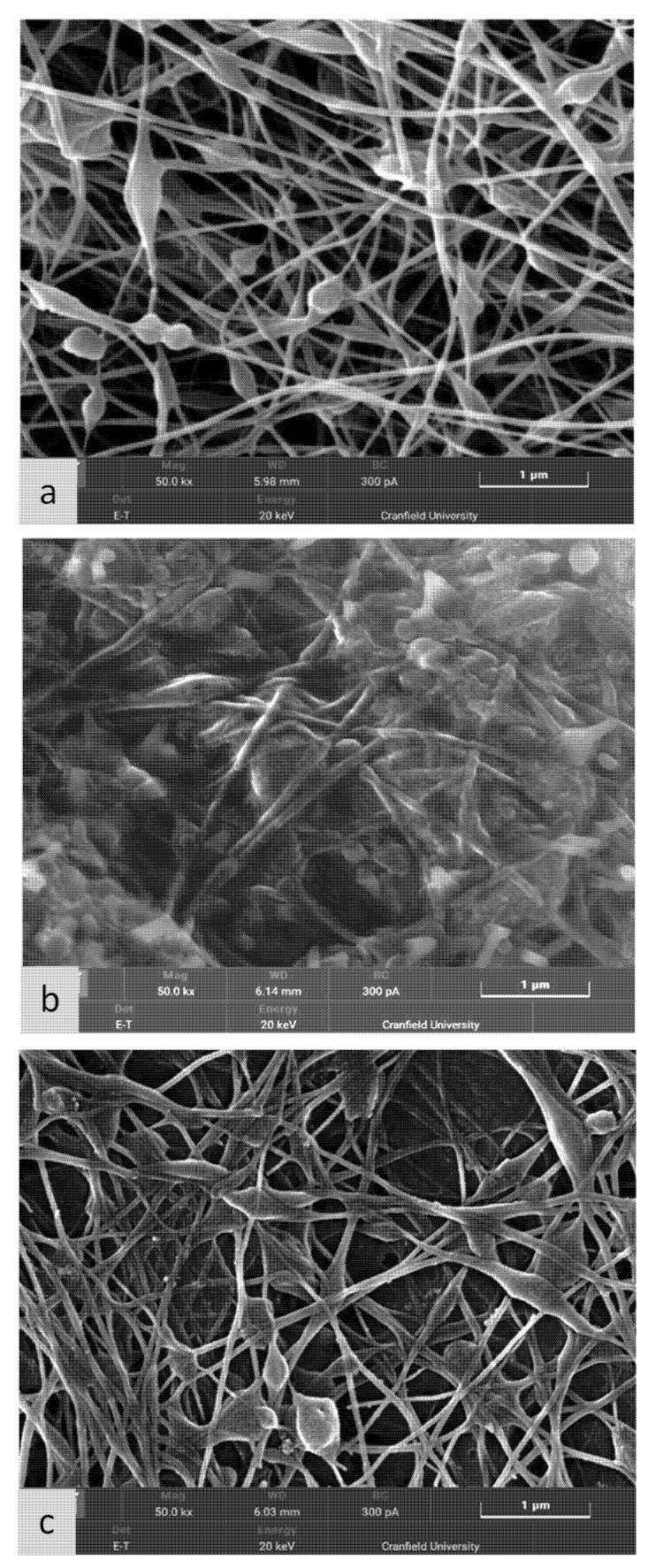
SEM of different samples with and without crosslinking: (**a**) S4—without crosslinking; (**b**) S5—after crosslinking with CaCl_2_, and (**c**) S6—after crosslinking with calcium chloride and water soak procedure for 24 h.

In this study, crosslinking was successfully developed, and in addition, different settings were modified to produce optimum nanofibre mesh. For crosslinking, calcium chloride was used, and three different samples were prepared, as shown in Figure 8 (also refer to Section 2.4 for experimental sample details). The process of adding reagents affected the electrospun fibres, as illustrated in the micrographs. The effect of crosslinking and the water soaking procedure is illustrated in Figure 8a–c. Therefore, at this stage, three types of samples were produced:

- Sample without crosslinking (referred to as S4);
- Sample after crosslinking for ten minutes using calcium chloride aqueous solution (referred to as S5);
- Sample after crosslinking with calcium chloride and soaked in deionised water for 24 h (referred to as S6).

As observed from Figure 8a, the SEM nanofibre structure of sample S4 was without crosslinking, and before soaking it with the aqueous crosslinking medium (calcium chloride solution), the fibre morphology was clear. However, the sample shown in Figure 8b was soaked in the aqueous crosslinking medium for ten minutes. The alginate was crosslinked with calcium chloride after exposure for ten minutes. However, PEO was not crosslinked with calcium chloride. Hence, PEO was leached out. This PEO leaching can be observed in Figure 8b, where the nanofibres are embedded in PEO film layers. After leaving the same sample in an aqueous medium (deionised water) for 24h, PEO was completely removed. Hence, we observed a clear nanofibre morphology, as shown in Figure 8c.

The alginate nanofibres are water soluble; they need a crosslinking procedure after the electrospinning process using ionic crosslinking solvent (calcium chloride), unlike synthetic polymers (polystyrene, polyvinyl chloride, (PVC), poly–lactic acid (PLA)), which do not need the crosslinking procedure following the electrospinning process. Therefore, our alginate nanofibre electrospinning process cannot be directly compared with synthetic nanofibre manufacturing (polystyrene, polyvinyl chloride, PVC, poly-lactic acid (PLA)).

The bead formation in our manufacturing process was a result of crosslinking processes. Even if we could reduce the formation of beads during the electrospinning process, by controlling the voltage and viscosity of the solution, during the crosslinking process, there would be a relatively smaller number of beads while maintaining a majority of nanofibres (Figure 8c). Hence, the drop-like bond formation leading to bead formation at the intersection of the nanofibres could not be completely avoided using the ionic crosslinking process.

The crosslinking process is necessary to prevent the alginate nanofibres from dissolving in water for their end-use. We obtained a majority of nanofibres with a relatively small number of beads. Overall, we showed that we could obtain ionically crosslinked nanofibres embedded with oregano oil, and such a nanofibre mat can be used for antimicrobial applications, which is sufficient for the proposed application in medical textiles (see Section 3.6).

### 3.4. Measurement of Nanofibre Diameter Distribution

After a series of parameter verification tests, a set of production processes that produced alginate fibre mesh with OEO with a good nanoscale microstructure were established, and mechanical properties were evaluated. S1 and S10 were both produced by this process. Due to the limitation of experimental conditions and sample size, it was not possible to perform multiple tests on a sample at the same time. Therefore, these two samples were used for measurements of diameter and mechanical tensile strength, respectively.

**Figure 9:**
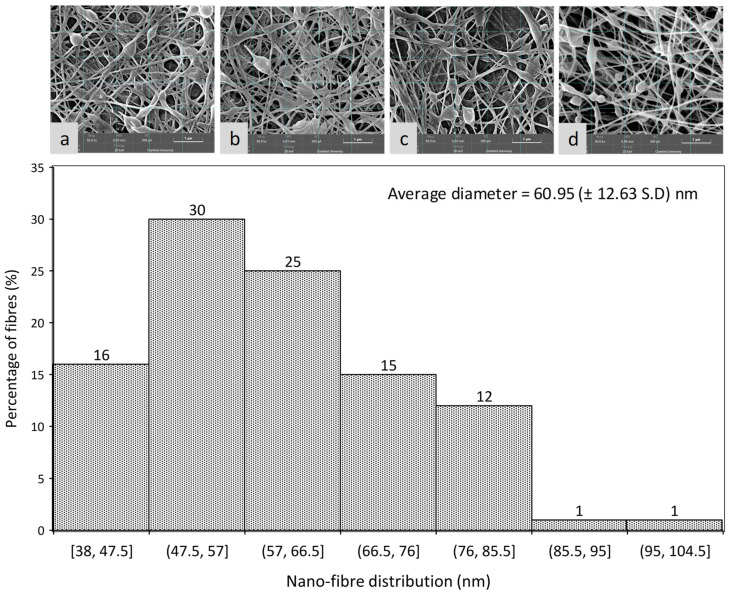
Nanofibre diameter distribution; SEM micrographs for sample S1; (**a**–**d**) different locations of S1 sample.

There were four-electron micrographs taken from different positions (Figure 9a–d), and these were used to count the fibre diameter (leading to 100 fibre diameter data sets). A histogram plot showed the variation of fibre diameter from 38–105 nm. According to these assays, there were 80–90% of fibres in the range of 38–78 nm in diameter, and approximately 50% of fibres were in the range of 48–68 nm in diameter. Because of the crosslinking reaction, there was a certain degree of adhesion between the fibres at the intersection. Likewise, there were still some beads (diameter around 200–400 nm) in the fibre bundle, but these were not enough to affect the overall appearance of the fibre structure.

### 3.5. Mechanical Testing of Alginate/OEO Fibre Mesh

**Figure 10:**
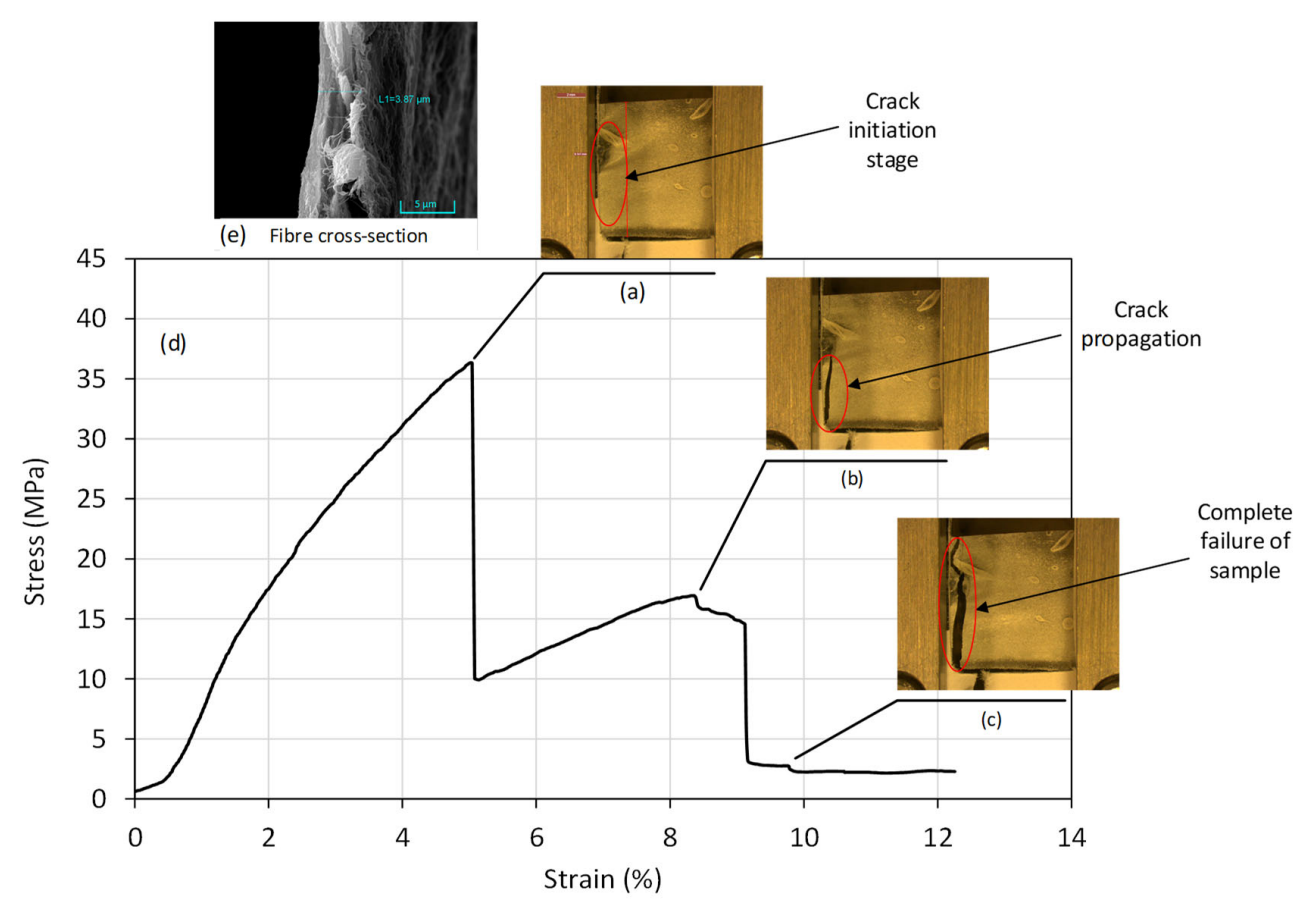
Mechanical testing of electrospun alginate/OEO fibre mesh: real-time close-up photos of sample (**a**) fibre mesh crack initiation, (**b**) crack propagation, and (**c**) complete failure of sample; (**d**) the stress–strain plot; (**e**) typical fibre cross-section.

Figure 10d illustrates a typical stress–strain curve of the sample (S10) with OEO. The highest peak value corresponds to tensile strength, which was found out to be 36 MPa. Figure 10a shows the stretched alginate/OEO nanofibre mesh. Further strain resulted in failure of material in three steps, as shown in Figure 10a–c. The final strain to failure of the nanofibre sample was 8.2%. Figure 10e illustrates a typical cross-section of the alginate/OEO mesh, with a thickness of 3.8 μm. Results indicated that the alginate/OEO mesh possessed sufficient strength and Young’s modulus (676.79 MPa; calculated in the region of 0.5–1.5% strain).

### 3.6. Antimicrobial Activity of Alginate Nanofibres

**Figure 11:**
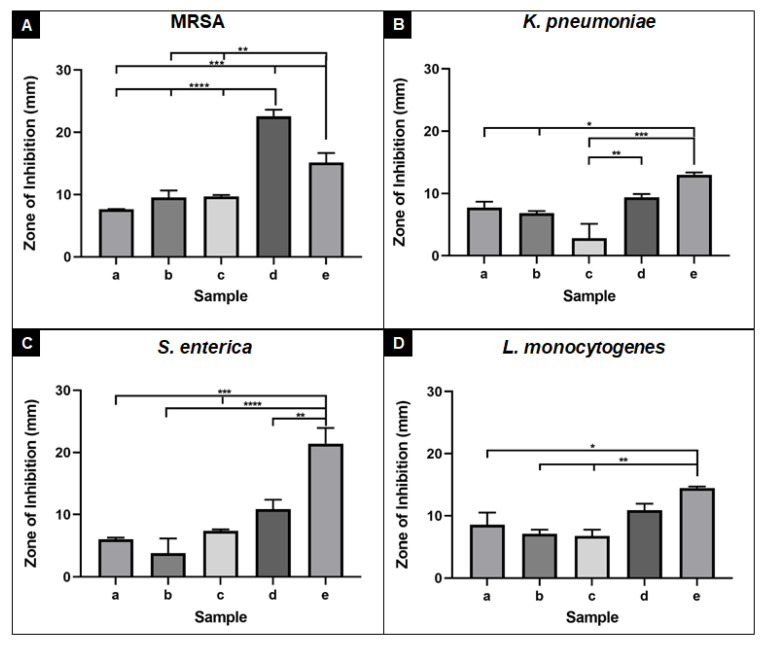
Antimicrobial efficacy of (a) with OEO (2%) before crosslinking, (b) with OEO (2%) after crosslinking, (c) with OEO (3%) crosslinked after water soak, (d) with additional OEO, (e) OEO control against (**A**) MRSA, (**B**) *K. pneumoniae*, (**C**) *S. enterica*, and (**D**) *L. monocytogenes*. Error bars represent standard error of the mean of *n* = 3 biological replicates. **** *p* ≤ 0.0001, *** *p* ≤ 0.001, ** *p* ≤ 0.01, * *p* ≤ 0.05.

Alginate nanofibre samples were subjected to antimicrobial contact assays against two common causes of bacterial wound infections (MRSA and *K. pneumoniae*) and two foodborne pathogens (*S. enterica* and *L. monocytogenes*). Nanofibres without OEO demonstrated no antimicrobial activity against all bacterial pathogens (data not shown). Upon addition of 3% OEO, nanofibres demonstrated antimicrobial efficacy by producing mean inhibitory zones between 6 and 8.5 mm against all four bacterial strains. When nanofibre samples were crosslinked, antimicrobial efficacy was retained and enhanced against MRSA (Figure 11A (b)). Similar levels of antimicrobial activity were observed when crosslinked samples were subjected to a water soak procedure (Figure 11A–D (c)). Nanofibres supplemented with additional OEO (0.02 mL/cm^2^) demonstrated enhanced antimicrobial activity against all four bacterial pathogens and was significantly more active against *K. pneumoniae* (*p* ≤ 0.01) compared to the crosslinked sample post water soak (Figure 11B, compare (d) with (c)). The additional OEO sample was also significantly more active against MRSA than all other alginate samples (*p* ≤ 0.0001) (Figure 11A, compare (d) with (a), (b), and (c)) and the OEO only control (*p* ≤ 0.01) (Figure 11A, compare (d) with (e)). Comparable activity was observed between the additional OEO nanofibres and OEO only control against *K. pneumoniae* and *L. monocytogenes*, indicating that the nanofibre technology represents a successful delivery system for OEO, which could be used in wound care management (dressings) or food packaging technology.

The chemical composition of OEO was reported in the previous research, which mainly consists of p-cymene (35.86%), carvacrol (28.85%), γ-terpinene (8.41%), and thymol (4.86%), [34]. These components are responsible for the antimicrobial behaviour of OEO [35,36]. The composition of OEO may vary depending upon the oregano growing conditions [37,38,39,40,41,42]. Future research will be conducted to determine the effect of OEO composition and the production methods to reduce the impact of harvest and growing conditions on the chemical composition of OEO.

## 4. Conclusions

In this study, we have shown the electrospinning of sodium alginate nanofibres incorporated with OEO. We have demonstrated a novel method of producing a clear alginate nanofibre morphology by carrying out a two-step crosslinking process. The first step gives us crosslinking of alginate, and the second step involving the use of deionised water soaking procedure for 24h gives a clear nanofibre morphology. Various process parameters (applied voltage and solution feed rate) were suitably optimised to ensure a stable Taylor cone formation was obtained. SEM analysis revealed alginate–OEO nanofibre diameter distribution between 38 and 105 nm. Mechanical tests revealed that alginate nanofibre mesh had adequate strength and thickness.

Antimicrobial assays showed that alginate nanofibres with incorporated OEO successfully inhibited the growth of clinically relevant Gram-positive and Gram-negative bacterial wound and foodborne pathogens. Notably, nanofibres containing OEO demonstrated greater antimicrobial efficacy (as determined by larger bacterial inhibitory zones) than OEO alone against multidrug-resistant MRSA bacteria, which is a major cause of bacterial healthcare-associated and wound infections [43]. Furthermore, the observed antimicrobial activity against both *S. enterica* and *L. monocytogenes*, the causative agents of foodborne illness, demonstrated a potential role for antimicrobial incorporated alginate nanofibre technology for use in food packaging. Overall, this study demonstrated that OEO could be incorporated within alginate nanofibres while maintaining physical and antimicrobial properties. Indeed, this study could potentially explore the successful delivery of antimicrobial medical textiles in biomedical applications and food packing applications.

## Figures and Tables

**Table 1:** Samples produced: preparation of solution for electrospinning and manufacturing process parameters.

Sample ID	Addition of OEO (Y/N)	Crosslinking with Calcium Chloride(Y/N)	Water Soak * (Y/N)	Applied Voltage (kV)	Notes
S1, S10	Y (2%)	Y	Y	30	Used for diameter measurement, mechanical test
S2	N	N	N	30	Influence of adding Oregano and viscosity
S3	Y (2%)	N	N	30	
S4	Y (2%)	N	N	26	Influence of different crosslinking process
S5	Y (2%)	Y	N	26	
S6	Y (2%)	Y	Y	26	
S7	Y (2%)	N	N	22	Influence of different spinning voltage
S8	Y (2%)	N	N	26	
S9	Y (2%)	N	N	30	

* Water soaking was carried out post-crosslinking process as an additional step.

## Data Availability

Data are contained within this article.
